# Selective Drain Placement in Complicated Laparoscopic Cholecystectomy: Indications, Intraoperative Determinants, and Outcomes

**DOI:** 10.7759/cureus.113160

**Published:** 2026-07-22

**Authors:** Rehan Farooq, Meenakshi Yeola

**Affiliations:** 1 Department of General Surgery, All India Institute of Medical Sciences, Mangalagiri, IND

**Keywords:** abdominal drain, bile leak, complicated laparoscopic cholecystectomy, drain placement, intraoperative drainage, parkland grading scale, postoperative outcomes

## Abstract

Background: The utility of intraoperative drain placement after complicated laparoscopic cholecystectomy (cLC) remains poorly defined. Present evidence strongly discourages the routine drainage after uncomplicated procedures and its role in high-risk intraoperative scenarios such as intraoperative contamination, bile leak, and others, which requires further characterization.

Objectives: This study aims to assess the utility of drains in detecting postoperative complications such as bile leaks and hemorrhage, and to evaluate their role and necessity in cLC.

Methods: A prospective descriptive longitudinal study was conducted over 18 months (January 2024-June 2025) at a tertiary care teaching institute. Seventy-six patients who underwent cLC with intraoperative drain placement meeting the Society of American Gastrointestinal and Endoscopic Surgeons (SAGES) criteria were enrolled. Demographic data, intraoperative findings (Parkland Grading Scale (PGS), critical view of safety (CVS) achievement, operative duration), indication for drain placement, daily drain output, visual analog scale (VAS) pain scores, and postoperative complications were recorded and analyzed using IBM SPSS Statistics for Windows, Version 21 (Released 2012; IBM Corp., Armonk, New York, United States).​​​​​​

Results: Mean age was 49.86 ± 13.27 years, with 44 (57.9%) female participants and a median hospital duration of four days. The most common indications for drain placement were hemorrhage risk, infection or contamination, and anatomical difficulty observed in 28 (36.8%), 21 (27.6%), and 13 (17.1%) patients, respectively. Parkland Grade III was the most prevalent intraoperative finding in 37 (48.7%) patients, with a CVS achieved in 67 (88.2%) of them. The CVS had a highly significant association with reason for drain placement (p < 0.001). Drain output was predominantly serosanguineous; bilious drainage occurred exclusively in the bile leak risk group, validating selective drain use. Precautionary drains were removed as per the standard recommendations (3-4 days), as compared to the risk-driven drains (hemorrhage, bile leak, infection) required retention for a duration of 5-7 days. VAS scores declined progressively from 6 to 1 on day 1 and day 6, respectively. One postoperative bile leak was identified and successfully managed by reexploration and transfixation of the blowout cystic duct stump with no other major postoperative complications.

Conclusion: Selective drain placement in cLC is clinically justified and effective. Drain output patterns correlated with the intraoperative indication, supporting an indication-guided approach to duration of drain retention. A tailored drain use aligned with the SAGES guidelines is recommended to improve patient safety by preventing morbidity.

## Introduction

Laparoscopic cholecystectomy (LC) is among the most frequently performed elective surgical procedures worldwide, including in India, where the prevalence of gallstone disease is estimated at 4.15%. Gender-wise distribution has been reported to be higher among females (5.59%) than among males (1.99%) [1]. It has well-documented advantages, including shorter hospitalization, faster recovery, reduced postoperative pain, and superior cosmetic outcomes compared with open surgical procedures, and has been the gold standard for the management of symptomatic cholelithiasis [2]. However, certain patient- and disease-related factors might substantially increase the procedural complexity. Clinical conditions such as acute or chronic cholecystitis, empyema, gallbladder perforation, mucocele, Mirizzi syndrome, and cholecystoenteric fistula elevate the technical challenge and the risk of adverse outcomes [3,4]. These complicated cholecystectomies carry a higher risk of intraoperative bleeding, bile spillage, and the need for advanced maneuvers or conversion to open surgery.

The surgeons often employ adjunctive strategies to mitigate postoperative risks. The selective placement of intraabdominal drains is one of these strategies. Abdominal drains are intended to detect and manage early postoperative complications such as bile leakage, infections, or hemorrhage. Though evidence from multiple systematic reviews and randomized controlled trials has discouraged routine drain use after uncomplicated LC [5], the Society of American Gastrointestinal and Endoscopic Surgeons (SAGES) endorses selective drainage under specific circumstances such as suspicion of bile duct injury, persistent intraoperative bleeding, extensive ooze from the gallbladder bed, difficult dissection, or common bile duct exploration [6]. Despite these guidelines, evidence supporting drain use in cLC is limited and inconsistent, especially in the South Asian region. Available published literature is mostly focused on uncomplicated procedures, and outcomes are not separated by specific intraoperative indications for drainage. Indication-specific data are needed to guide decisions in high-risk scenarios. This study was designed to evaluate the clinical utility of selective intraoperative drain placement in cLC at a tertiary teaching institute, aiming to check the correlation between indication for drainage, drain output characteristics, and postoperative outcomes.

## Materials and methods

Study design and setting

This prospective descriptive longitudinal study was conducted in the Department of General Surgery at All India Institute of Medical Sciences (AIIMS), Mangalagiri, a tertiary care teaching hospital in Andhra Pradesh, India. The study enrolled patients over an 18-month period from January 2024 to June 2025. The Institutional Ethics Committee (IEC) of AIIMS, Mangalagiri, India, approved this study (AIIMS/MG/IEC/2023-24/54). The following variables were systematically documented for each participant. Demographic details included age, sex, comorbidities (diabetes mellitus, hypertension), and others. Intraoperative findings included primary diagnosis, Parkland Grading Scale (PGS) [7], achievement of the critical view of safety (CVS), operative duration, and type of cholecystectomy (total vs. subtotal). Drain-related variables studied were type and reason for drain placement, daily drain output (volume in mL and character: serous, serosanguineous, bilious, or purulent), along with drainage duration. The postoperative outcomes collected were visual analog scale (VAS) pain scores recorded from day 1 to 7, bile leak, intraabdominal collections, surgical site infection (SSI), and hospital stay duration.

Inclusion and exclusion criteria

All the patients undergoing complicated laparoscopic cholecystectomy (cLC), who met the SAGES criteria for intraoperative drain placement, including suspicion of bile duct injury, difficult gallbladder dissection, persistent intraoperative bleeding, extensive gallbladder bed ooze, intraoperative cholangiography, presence of infected bile leak, or common bile duct exploration, and willing to give consent were included. The patients undergoing uncomplicated LC without intraoperative complications; patients who did not satisfy the SAGES criteria for drain placement; cases converted to open cholecystectomy; and patients who declined consent were excluded from this study.

Sample size

Sample size was calculated using the formula "$n=\frac{4pq}{d^2}$", assuming an expected proportion of 60% of complicated cases requiring drain placement (p = 0.6, q = 0.4), a relative margin of error of 20% (d = 0.12), and a 10% attrition allowance, with total patients (n) calculated to 76. All 76 patients treated during the study period were included in the final analysis.

Assessment tools

PGS 

An intraoperative classification system ranging from Grade I (normal-appearing gallbladder) to Grade V (severe inflammation with perforation, gangrene/necrosis, or inability to visualize the gallbladder). Grade II marked minor adhesions at the gallbladder neck; Grade III showed the presence of hyperemia or pericholecystic fluid; Grade IV had adhesions obscuring most of the gallbladder or abnormal anatomy. This scale was used to standardize intraoperative assessment and guide decision-making [7].

VAS

A validated 10-point unidimensional pain scale (0, no pain; 10, worst pain) was used to assess postoperative pain intensity at specified intervals from the 1st to 7th day [8].

Drain Output Monitoring

Daily drainage volume was measured in milliliters from the closed-system drainage container over the preceding 24 hours. Color and consistency were noted qualitatively. Measurements were performed by trained staff under supervision to minimize interobserver variability.

Statistical analysis

All statistical analyses were performed using IBM SPSS Statistics Version 21. Descriptive statistics were used to characterize the study population. Normally distributed continuous variables are reported as mean ± standard deviation (SD); nonnormally distributed variables as median with interquartile range. Categorical variables are reported as frequencies and percentages. Associations between categorical variables were evaluated using the Pearson chi-square test. Normality was assessed by the Shapiro-Wilk test prior to the selection of parametric or nonparametric tests. A two-tailed p-value of <0.05 was considered statistically significant.

## Results

Demographic and clinical profile

A total of 76 patients who underwent cLC with intraoperative drain placement were enrolled over the period of 18 months. The median age was 49.00 years (range: 22-86 years). The majority of the study cohort was observed in the fifth decade of life. A total of 44 (57.9%) of the cohort were females. When stratified by sex, female patients had a mean age of 46.95 ± 12.43 years versus 53.84 ± 13.55 years in males, indicating a tendency for older age at presentation in male patients (Table 1).

**Table 1:** Demographic profile of study participants SD: standard deviation; n: number; %: percentage

Variable	Value
Age (years) (mean ± SD)	49.86 ± 13.27
Female (n/%)	44 (57.9%)
Male (n/%)	32 (42.1%)
Diabetes mellitus (n/%)	30 (39.5%)
Hypertension (n/%)	26 (34.2%)

Intraoperative findings

The PGS distribution showed Grade III with 37 (48.7%) patients as the most prevalent finding, followed by 22 (28.9%) patients in Grade IV. No Grade I cases were included, consistent with the complicated nature of the cohort. CVS was achieved in 67 (88.2%) of 76 cases. A highly significant association was found between the reason for drain placement and achievement of CVS (χ² = 26.909; df = 4; p < 0.001), with bile leak risk cases being least likely to achieve CVS (Table 2).

**Table 2:** Intraoperative findings by Parkland Grading Scale classification

Parkland Grade	Frequency (n)	Percentage (%)
Grade II	14	18.4
Grade III	37	48.7
Grade IV	22	28.9
Grade V	3	3.9
Total	76	100.0

Indications for drain placement

The distribution of indications for intraoperative drain placement is presented in Table 4. Hemorrhage risk was the most frequent indication with 28 (36.8%) patients, followed by infection/contamination with 21 (27.6%) patients. The indications for drain placement in relation to achievement of the CVS. Drains were inserted in nine of 76 patients (11.84%), mainly in cases with a perceived risk of bile leak, difficult dissection, hemorrhage, or infection/contamination. The association between the indications for drain placement and CVS status was statistically significant (Pearson χ² = 26.909; df = 4; p < 0.001), indicating that drains were predominantly used in higher‑risk or technically challenging cases (Table 3).

**Table 3:** Association between reason for drain placement and achievement of the critical view of safety CVS: critical view of safety; df: degree of freedom

Drain placement indication	Total cases, n (%)	CVS achieved, n (%)	CVS not achieved, n (%)
Anatomical difficulty/variants	13 (17.10)	13 (19.40)	0 (0.00)
Bile leak risk	8 (10.52)	3 (4.48)	5 (55.56)
Difficult dissection	6 (7.89)	4 (5.97)	2 (22.22)
Hemorrhage risk	28 (36.84)	27 (40.30)	1 (11.11)
Infection/contamination	21 (27.63)	20 (29.85)	1 (11.11)
Total	76 (100)	67 (88.16)	9 (11.84)
Pearson χ²	26.909		
df	4		
p-value	<0.001		

Drain removal

The majority of drains were removed by postoperative day (POD) 2 and 3, with 23 (30.67%) and 26 patients (34.67%), respectively, summing up to 49 patients (65.33%), except one patient who underwent surgical reexploration for postoperative bile leak. Precautionary drains (anatomical difficulty, difficult dissection) were removed by 3-4 days, while risk-driven drains (bile leak, hemorrhage, infection) required retention until 5-7 days, guided by output trends and clinical suspicion.

Postoperative outcomes

VAS pain scores declined progressively across all patients, from a mean of 6 on POD 1 to approximately 1 on POD 6, indicating effective analgesic management and resolution of acute postsurgical pain. Median hospital stay for the cohort was four days. The majority of patients (75/98.68%) did not experience any postoperative complications (hemorrhage requiring intervention, surgical site infection, or intraabdominal collection), except one patient who developed a postoperative bile leak. This bile leak was identified by monitoring the drain output characteristics (bilious drainage confirmed in the bile leak risk group) and was managed with timely reexploration and transfixation of the blowout cystic duct stump. No mortality was recorded.

## Discussion

The role of prophylactic drain placement after laparoscopic cholecystectomy has been extensively investigated, with consistent evidence discouraging its routine use in uncomplicated cases due to lack of benefit and potential for increased morbidity [9,10]. However, in the setting of cLC characterized by advanced inflammation, dense adhesions, intraoperative bile spillage, hemorrhage risk, or subtotal procedures, there is limited evidence, which is largely qualitative. The present study provides systematic, indication-stratified data on drain utility in this high-risk cohort. The demographic profile of our cohort, with a mean age of approximately 50 years and a female predominance with 44 (57.9%) female patients, was consistent with the known epidemiology of gallstone disease in South Asia, where women are disproportionately affected [1]. The dominance of Parkland Grade III pathology (n = 37; 48.68%) reflects a moderate-to-severe gallbladder inflammation as the primary driver of surgical complexity in this research study.

Hemorrhage risk was noted in 28 (36.8%) of the study patients and was observed as the leading indication for drain placement in this study setting. This was consistent with the data reported by Lee et al., a study that studied the intraoperative risk factors as important determinants of selective drainage [11]. Hemorrhage risk was followed by infection/contamination and anatomical difficulty, which were observed in 21 (27.6%) and 13 patients (17.1%), respectively. These findings were similar to the studies identifying operative complexity and intraoperative risk factors as major determinants of drain placement [12]. Bile leak risk justified selective drainage in a smaller but clinically important subgroup. Although routine drainage is not supported by current evidence, selective use in patients perceived to be at higher risk for postoperative complications remains a common surgical practice [13]. Difficult dissection was the least common indication, with only six patients (7.9%) affected. These patients were managed as per the SAGES recommendations supporting selective drain placement in situations involving difficult dissection, uncertain anatomy, or increased risk of postoperative complications [5].

The results of this research showed a highly significant association between the reason for drain placement and achievement of CVS (p < 0.001). The patients with drainage for bile leak risk were the most likely to fail CVS achievement. This underscores the importance of the relationship between anatomical obscurity, operative difficulty, and the need for postoperative surveillance. This finding has practical implications: failure to achieve CVS should be considered an independent trigger for drain placement consideration. The drain output profiles in this study revealed bilious drainage exclusively in the bile leak risk subgroup. Only a single patient had postoperative bile leak in this study group and was successfully detected through drain monitoring and subsequently managed with timely surgical intervention. This demonstrates the sentinel function of drains in high-risk patients as not merely prophylactic but might be utilized for its potential to be actively used for diagnostic purposes.

Serosanguineous output predominated in hemorrhage and infection groups, with the gradual transition to serous drainage guiding safe removal timing. Precautionary drains (anatomical difficulty, difficult dissection) were cleared early, enabling removal for three to four days' duration, while risk-driven drains required a longer retention period (5 to 7 days). The observed indication-specific differences in retention duration advocate for a tailored removal strategy rather than uniform early- or late-removal policies. The finding that 49 (65.33%) patients' drains were removed by day 2 and 3 was similar to the published literature advocating early drain removal to reduce SSI risk [14]. VAS pain scores declined steadily from six points on day 1 to one point by day 6, suggesting that the presence of drains did not substantially impair pain control in this complicated cohort, a contrast to findings in uncomplicated LC studies where drain presence was associated with elevated pain scores [15,16]. The median hospital stay of four days compares favorably with reported stays in cLC series from similar-resource settings, suggesting that selective drain use did not prolong hospitalization when combined with early removal protocols. The absence of major postoperative complications in this cohort is notable and may reflect the effectiveness of the perioperative care pathway at a tertiary teaching institute. However, it also highlights a recognized limitation of single-arm studies: without a comparator group of patients without drains in comparable complicated scenarios, the independent contribution of drainage to complication prevention cannot be quantified.

Limitations

This study has several limitations. First, the absence of a control (no-drain) arm precludes causal inference about the benefit of drainage over nondrainage in cLC. Second, convenient sampling may introduce selection bias. Third, the single-center design limits generalizability to other institutional settings, particularly those with differing case complexity profiles or surgical expertise. Fourth, the study period captured a specific institutional experience, and outcomes may reflect local surgical culture and learning curve effects. Future multicenter randomized controlled trials stratified by indication for drainage are needed to establish definitive guidelines.

## Conclusions

The drains were placed selectively in patients judged to be at higher intraoperative risk (such as suspected bile leak, difficult dissection, hemorrhage, or contamination) and enabled early detection of a bile leak in one patient in this observational research study. The nature and duration of drain output correlated well with the intraoperative indication for drainage, supporting an evidence-based, indication-guided approach to drain retention. Drains served an important sentinel function in the bile leak risk subgroup, enabling early detection and management of the sole postoperative bile leak. These findings support tailored, SAGES-aligned drain use in high-risk gallbladder surgeries to optimize patient safety while avoiding unnecessary morbidity. Larger, multicenter studies with comparator groups are recommended to further validate these results and develop standardized indications and duration protocols for drain placement in cLC.
